# Sequential Loading of Cohesin Subunits during the First Meiotic Prophase of Grasshoppers

**DOI:** 10.1371/journal.pgen.0030028

**Published:** 2007-02-23

**Authors:** Ana M Valdeolmillos, Alberto Viera, Jesús Page, Ignacio Prieto, Juan L Santos, María Teresa Parra, Margarete M. S Heck, Carlos Martínez-A, José L Barbero, José A Suja, Julio S Rufas

**Affiliations:** 1 Departamento de Biología, Edificio de Biológicas, Universidad Autónoma de Madrid, Madrid, Spain; 2 Department of Immunology and Oncology, Centro Nacional de Biotecnología, Madrid, Spain; 3 Departamento de Genética, Facultad de Biología, Universidad Complutense, Madrid, Spain; 4 Wellcome Trust Centre for Cell Biology, Institute of Cell Biology, University of Edinburgh, Edinburgh, United Kingdom; 5 Departamento de Biología Celular y del Desarrollo, Centro de Investigaciones Biologicas (CSIC), Madrid, Spain; Stowers Institute for Medical Research, United States of America

## Abstract

The cohesin complexes play a key role in chromosome segregation during both mitosis and meiosis. They establish sister chromatid cohesion between duplicating DNA molecules during S-phase, but they also have an important role during postreplicative double-strand break repair in mitosis, as well as during recombination between homologous chromosomes in meiosis. An additional function in meiosis is related to the sister kinetochore cohesion, so they can be pulled by microtubules to the same pole at anaphase I. Data about the dynamics of cohesin subunits during meiosis are scarce; therefore, it is of great interest to characterize how the formation of the cohesin complexes is achieved in order to understand the roles of the different subunits within them. We have investigated the spatio-temporal distribution of three different cohesin subunits in prophase I grasshopper spermatocytes. We found that structural maintenance of chromosome protein 3 (SMC3) appears as early as preleptotene, and its localization resembles the location of the unsynapsed axial elements, whereas radiation-sensitive mutant 21 (RAD21) (sister chromatid cohesion protein 1, SCC1) and stromal antigen protein 1 (SA1) (sister chromatid cohesion protein 3, SCC3) are not visualized until zygotene, since they are located in the synapsed regions of the bivalents. During pachytene, the distribution of the three cohesin subunits is very similar and all appear along the trajectories of the lateral elements of the autosomal synaptonemal complexes. However, whereas SMC3 also appears over the single and unsynapsed X chromosome, RAD21 and SA1 do not. We conclude that the loading of SMC3 and the non-SMC subunits, RAD21 and SA1, occurs in different steps throughout prophase I grasshopper meiosis. These results strongly suggest the participation of SMC3 in the initial cohesin axis formation as early as preleptotene, thus contributing to sister chromatid cohesion, with a later association of both RAD21 and SA1 subunits at zygotene to reinforce and stabilize the bivalent structure. Therefore, we speculate that more than one cohesin complex participates in the sister chromatid cohesion at prophase I.

## Author Summary

Meiosis is a specialized cell division by which sexually reproducing organisms prompt the formation of specialized cells presenting a half of the species chromosomal number. These cells, the so-called gametes, are able to fertilize or be fertilized, depending on the sex in which they are produced and thus restore the species chromosomal number after fertilization. The reduction in the chromosome number is achieved by two successive rounds of chromosome segregations preceded by a single replication of the genetic material. Different proteins, mainly referred to as cohesins, are implied in the correct establishment and maintenance of an intimate association between homologous chromosomes by ensuring their close association until their separation in the first meiotic division. Grasshoppers have been considered as a gorgeous model for meiotic studies for decades due to their low chromosomal number, the large size of their chromosomes, and the well-defined meiotic stages at cytological level. On these grounds, we have combined classical grasshopper chromosome knowledge with protein immunolocalization tools in order to precisely analyze the presence of cohesins throughout the prophase of the first meiotic division. The results not only describe the dynamic loading pattern of several cohesin subunits in two grasshopper species, but they also surprisingly bring into light that different cohesins are sequentially loaded onto meiotic chromosomes throughout the first meiotic prophase. Finally, we discuss the possible roles for this sequential protein loading in relation to the processes that operate during meiosis, proposing a model for meiotic chromosome structure. Besides the novel scientific contributions for a better understanding of the meiotic process, this study clearly points out that classical cytogenetic models can be used to solve modern biological problems.

## Introduction

Duplication of genetic material and its proper transmission to daughter cells must be scrupulously regulated in order to avoid errors that could modify the chromosomal complement of the species leading to aneuploidies. For this purpose, cells invariably achieve a round of DNA replication before each nuclear division when duplicated genomes separate into two identical cells. To assure a correct distribution, the previously duplicated DNA molecules must be joined from the time of their replication until their segregation at anaphase. The tight association between sister chromatids along their entire length is established by the mitotic cohesin complex. The general features of this complex are conserved from yeast to human [1,2]. This complex is mainly composed of four subunits: a heterodimer of two structural maintenance of chromosome proteins (SMC1 and SMC3), associated with two non-SMC components corresponding to sister chromatid cohesion proteins (SCC1 and SCC3) [3–6]; and for review see [7,8]. The SCC1 subunit is also called a mitotic chromosome determinant (MCD1) in Saccharomyces cerevisiae [9] or radiation-sensitive mutant (RAD21) in Schizosaccharomyces pombe [10]. In addition, in vertebrates there are two SSC3 subunits, which were first characterized as stromal antigen proteins (SA1 and SA2) [6,11].

Recent studies suggest that the association of the cohesin complex with chromatin and the following establishment and maintenance of cohesion are functionally separable, and that additional specific factors are required for each process to be achieved [10,12,13] (and for review see [14]). Proper sister chromatid cohesion may be established most efficiently during S phase since the two replicated sister DNA strands are closely apposed [12]. Cohesin-mediated connections are created at centromeres and at regular intervals along chromatid arms [15–18]. Several proteins are involved in the establishment of sister chromatid cohesion [19–22], despite that they may also be involved in other cellular functions [23–26]. The maintenance of cohesion throughout G2 is thought to facilitate the efficient repair of DNA double-strand breaks by homologous recombination between sister chromatids [27,28]. Furthermore, the cohesin complex plays an essential role ensuring bipolar attachment of sister chromatids to microtubules [21]. Therefore, the establishment of proper cohesion and its regulation during the cell cycle are of fundamental importance for genome stability.

In meiosis, a single DNA replication event precedes two consecutive rounds of chromosome segregation. In this process, cohesin complex not only maintains cohesion along the length of sister chromatids until the first meiotic anaphase, but also contributes to centromere cohesion up to the second meiotic anaphase. Additionally, this complex supports the interactions between homologous chromosomes and between sister chromatids during the initiation of recombination at prophase I [29] and plays a role in the maintenance of chiasmate bivalents until metaphase I [30–32]. Due to the variety of particular meiotic chromosome processes in which cohesion is implicated, it can be expected that meiocytes may contain distinct molecular complexes in order to ensure the specific behavior of chromosomes [33,34]. At present, it is well established in a variety of species that during meiosis some of the canonical mitotic subunits of the cohesin complex [33,35,36] coexist with several meiosis-specific variants such as meiotic recombination proteins (REC8 and REC11), Stromal antigen 3 (STAG3), and SMC1β [29,37–39] (for reviews see [40,41]).

The analysis of the temporal expression and loading of cohesin subunits onto meiotic chromosomes can allow us to elucidate the existence of different cohesin complexes and their role in the dynamics and structure of meiotic bivalents. For this purpose, we have performed these analyses in grasshopper males because, although these organisms have been classically used to analyze meiosis under a cytological point of view, there are no data regarding the participation of the cohesin complex and its loading dynamics in the meiotic chromosome organization. Taking into account the advantage of a certain degree of evolutionary conservation of the cohesin subunits among species, we have tested in two grasshoppers, Eyprepocnemis plorans and *Locusta migratoria,* several antibodies previously generated against human SMC3 and *Drosophila* cohesin subunits (DSA1 and DRAD21). The expression pattern of these three proteins has been analyzed on squashed spermatocytes, since this procedure preserves the structure and volume of the nucleus, allowing an accurate analysis of the 3-D relationships among them [42,43].

We have found that, whereas the cohesin axes defined by SMC3 allowed us to infer the position of both the axial and lateral elements (AEs/LEs) of the synaptonemal complex (SC) from preleptotene onward, the non-SMC cohesin subunits RAD21 and SA1 are not loaded onto chromosomes until their synapsis at zygotene. We propose that this second round of cohesin subunit loading reinforces the cohesion in the bivalent until its segregation at anaphase I. Finally, a possible model for the loading of cohesin subunits and the structure of meiotic chromosome during the first meiotic prophase is proposed and discussed.

## Results

### SMC3, SA1, and RAD21 Homologs in the Grasshopper Testis

**Figure 1:**
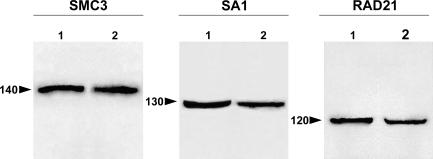
Immunoblotting Immunoblot analyses of the polyclonal antibodies against SMC3, SA1, and RAD21 in grasshopper testis extracts from L. migratoria (2 in the SMC3 and RAD21 panels) and E. plorans (2 in the SA1 panel). Nuclear extracts of mouse testis (1 in the SMC3 panel) and *Drosophila* Schneider cells (1 in the SA1 and RAD21 panels) are used as a control. The molecular mass markers are indicated by numbers and their position by arrowheads. Each antibody specifically recognized a single protein band in both control and grasshopper testis extracts.

To determine the immunoreactivity and specificity of anti-hSMC3, anti-DSA1, and anti-DRAD21 polyclonal antibodies in the species analyzed here, E. plorans and *L. migratoria,* we performed immunoblot analyses of grasshopper testis nuclear fractions. Mouse nuclear testis fraction and nuclear extract of Schneider cells from *Drosophila* were used as positive controls (Figure 1). In the grasshopper testis, each antibody specifically recognized a single band, all of them representing a similar molecular weight to that detected in the positive control extracts. The molecular weights of the immunoreactive bands in both grasshopper species and in the corresponding control were around 140 kDa for SMC3, 130 kDa for SA1, and 120 kDa for RAD21. Therefore, the antibodies used in the present study allowed us to identify, in grasshopper testis, the homologs of the cohesin subunits SMC3, SA1, and RAD21.

### SMC3 Cohesin Axis Maturation Reflects Synapsis Progression

**Figure 2:**
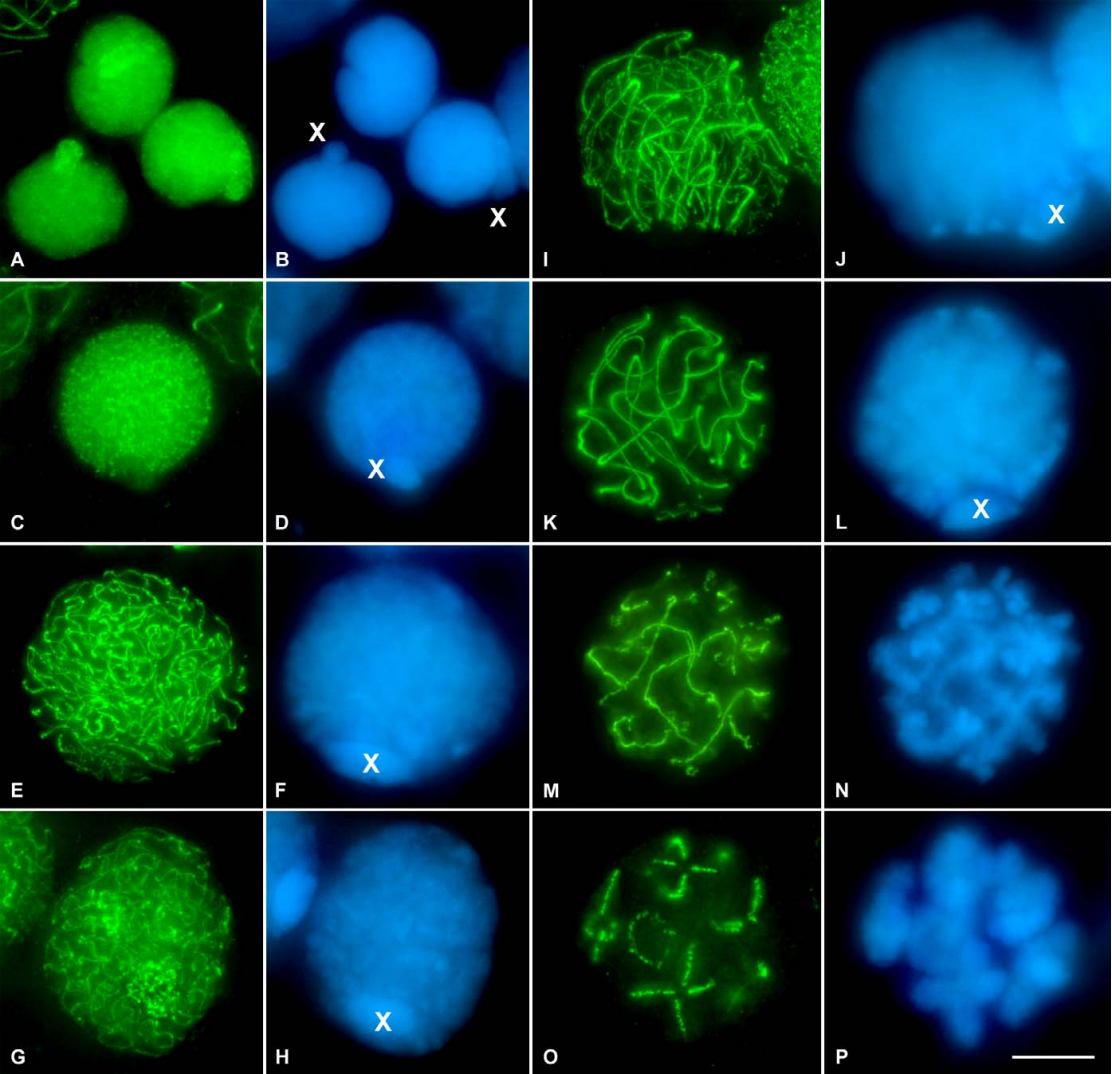
SMC3 Location in Spermatogonial Cells and in First Meiotic Prophase in the Grasshopper *L. migratoria* Spermatocytes (A and B) SMC3 is uniformly scattered throughout the nuclei of the spermatogonial cells, with the exception of the periphery of the single X chromosome. Note that the X chromosome is located into a nuclear protrusion. (C and D) In this preleptotene spermatocyte, SMC3 appears forming thin cohesion axis threads. Note the absence of labeling in the X chromosome. (E and F) In leptotene, there is a continuous formation of thin and single SMC3 treads, uniformly distributed into the entire nucleus. Note that the width of the X chromosome SMC3 axis is similar to those present in the autosomes. (G and H) The onset of zygotene in grasshopper spermatocytes is characterized by the presence of one or two regions of synapsis initiation. These synapsed regions are clearly seen due to the doubleness of the SMC3 threads width. (I and J) Zygotene spermatocyte in which the “bouquet” configuration is evident. (K and L) Pachytene spermatocyte characterized by the complete synapsis of autosomes denoted by the double width of the SMC3 lines. A thinner axial signal is present in the unsynapsed X chromosome univalent. (M and N) Early diplotene, in which desynapsis is accompanied by a barbed wire–like aspect of the SMC3 lines. (O and P) Late diplotene spermatocyte in which SMC3 is located at the interchromatid domain. It is interesting to note that no signals are found at the centromere regions. (B, D, F, H, J, L, N, and P) correspond to the DAPI-stained chromatin of the spermatocytes. The position of the single sex chromosome is marked with an X. 3-D reconstructions of all the cells of this plate are available in Video S1–S8.

The immunolocalization of the cohesin subunit SMC3 in squashed grasshopper testis preparations of the species analyzed (Figure 2) revealed a uniform pattern of small puncta over the spermatogonial nuclei (Figure 2A). These cells were easily distinguished due to an evident nuclear protrusion which corresponds to the single sex chromosome (Figure 2A and 2B). It is important to note that the sex chromosomal determination system in these grasshopper species is of the type XX for females, and XO for males; therefore, in males the X chromosome remains as a univalent throughout all meiotic stages. The labeling pattern of SMC3 on the chromatin of the X chromosome in spermatogonia is similar to that present in the rest of the autosomes (Figure 2A and Video S1). Preleptotene spermatocytes display a pattern of larger, albeit homogeneously distributed foci of SMC3 immunostaining in the nuclei, except at the X chromosome (Figure 2C and 2D and Video S2). At this stage, the X chromosome, which usually appears in the nuclear periphery (Figure 2D), exhibits a weaker and more diffuse SMC3 staining than that displayed by the autosomes (Figure 2C and 2D and Video S2). Leptotene spermatocytes show irregular discrete threads of SMC3, which appear to be continuous throughout the nuclear volume, denoting cohesin axis maturation (Figure 2E and Video S3). The peripherally located X chromosome (Figure 2F) presents a single SMC3 axis, which resembled in conformation and localization the AE observed in this chromosome under electron microscopy [44]. At the leptotene-zygotene transition, and concomitant with the onset of synapsis, it becomes evident that cohesin axes start to associate in pairs, forming thick filaments in one or two discrete nuclear regions (Figure 2G and Video S4). In zygotene spermatocytes, both paired and unpaired SMC3 axes are discernible as synapsis proceeds (Figure 2I and 2J). Additionally, we observe that all the cohesin axis ends congregate in a discrete nuclear region where they polarize in a *bouquet*-like arrangement (Figure 2I and Video S5). The single unpaired SMC3 axis of the X chromosome was also polarized into the bouquet configuration (Figure 2I and 2J and Video S5). At pachytene, cohesin axes achieve pairing at their full length, except the X chromosome, which remains unsynapsed (Figure 2K and 2L and Video S6). Hence, at this stage, the number of thick, paired axes corresponds in number and length to the 11 autosomal bivalents of the analyzed species (Figure 2K). All of the cohesin axis ends are distributed at the nuclear periphery where they seem to be associated to the nuclear envelope (this situation is clearly detected in 3-D reconstructed cells, as in the pachytene spermatocyte shown in Video S6). Once again, the X chromosome appears located in the nuclear periphery and displays a single unsynapsed axis, around half of the width of the paired axes present in autosomal bivalents (Figure 2K and Video S6).

### SMC3 Persists after Desynapsis between Homologous Chromosomes

In diplotene, desynapsis between homologs becomes evident after analyzing the DAPI staining of the spermatocytes (Figure 2N). This stage is characterized by the irregular appearance of the SMC3 cohesin axes, which present a barbed wire–like organization, with multiple excrescences running from the axes to the surrounding chromatin (Figure 2M and Video S7). From late diplotene onward, the SMC3 signals appear located in the bivalents at the so-called interchromatid domain [45] and also between the sister chromatids of the X chromosome (Figure 2O and Video S8).

### Anti-DSA1 Does Not Render Signal in Condensed Spermatogonial Chromosomes

In interphase spermatogonial cells, the anti-DSA1 antibody renders a weak uniform labeling, whereas in pachytene spermatocytes, lines resembling the structure of SCs are detected. In spermatogonial prophases, as chromatin condensation progresses, an increase of the labeling in the nuclear regions far apart from the chromosomal territories is observed. In these cells, centrioles and pericentriolar material are more intensively labeled. Cell poles are detected in all spermatogonial mitotic stages. Metaphase cells show bright protoplasm, but the chromosomes appear negatively labeled. It is interesting to note that no signaling is located either in the centromeric regions or between sister chromatids. This absence of labeling in the chromosomes is maintained until telophase. However, we have recently obtained results in the species *Chorthippus jucundus,* which indicated the absence of labeling in between chromatids despite the fact that SA1 is present at the centromeric region of spermatogonial-condensed chromosomes (unpublished data)

### SA1 Is Not Detected Over the Chromosome Axis at Early Meiotic Stages

**Figure 3:**
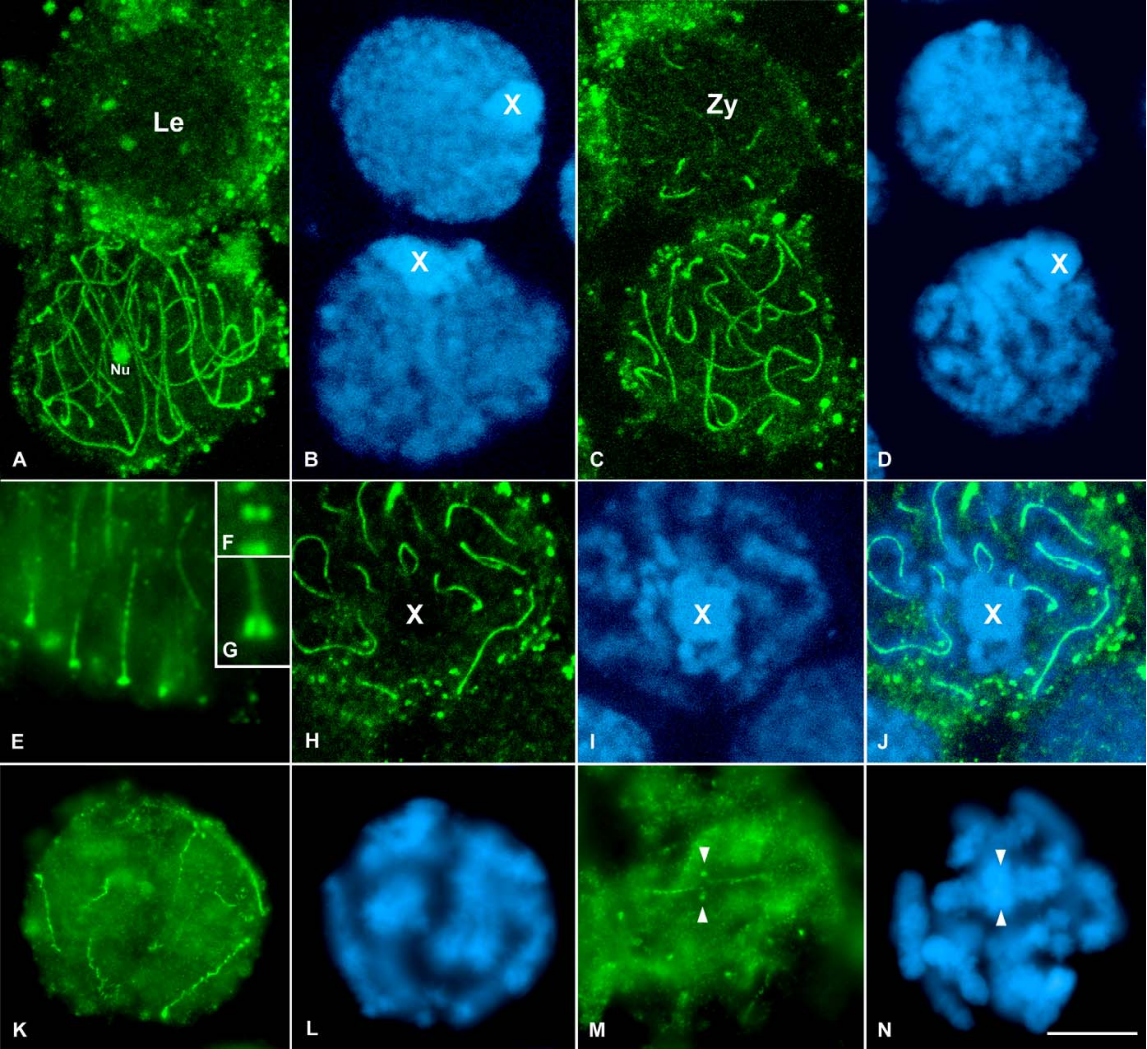
SA1 Location in the Grasshopper E. plorans First Meiotic Prophase Spermatocytes (A and B) Leptotene and pachytene spermatocytes. Note that, despite the fact that this image corresponds to the proyection of several focal planes, there is no SA1 signaling in the leptotene spermatocyte. Le, leptotene; Nu, nucleolus. (C and D) Zygotene and pachytene spermatocytes. Note the presence of short SA1 threads in the zygotene nucleus. Zy, zygotene. (E) Magnification of the periphery of a pachytene nucleus. Note the accumulation of SA1 at the ends of the linear structures formed by SA1 at their contact with the nuclear envelope. (F and G) Enlargements of this association in frontal (F) and lateral (G) views, respectively. (H and I) Projection of all focal planes throughout the univalent sex chromosome from a pachytene spermatocyte. The chromatin of this chromosome is easily distinguished. Note the absence of SA1 signal inside the sex chromosome. (J) Merged image of the SA1 staining and the chromatin counterstaining. The presence of SA1 signaling in the autosomes is quite evident. (K and L) Early diplotene cell in which a barbed wire–like staining of SA1 is seen. (M and N) Late diplotene spermatocyte showing SA1 staining at the interchromatid domain. Note the SA1 accumulations present at the homologous centromere regions of the bivalents (arrowheads). (B, D, I, L, and N) correspond to the DAPI-stained chromatin of the spermatocytes. The position of the single sex chromosome is marked with an X. (A, B, C, D, H, I, and J) are images from confocal microscopy. (E, F, G, K, L, M, and N) are images from fluorescence microscopy.

The immunolocalization of SA1 in grasshopper squashed spermatocytes (Figure 3) revealed that in leptotene cells, no appreciable amounts of SA1 labeling are present inside the nucleus, with the exception of nucleoli (Figure 3A and 3B). However, dispersed foci are detected along the nuclear periphery (Figure 3A and 3B). These signals appear paired and polarized in one discrete nuclear region at later meiotic stages. We interpret these foci as the visualization of the attachment plates of the SC due to their staining characteristics with this antibody (see Figure 3E–3G) and their pairing and polarization as the development of a bouquet-like configuration. At this stage, no SA1 label is detected on chromosome axis.

### SA1 Locates Over the Synapsed Regions of Chromosomes

In zygotene nuclei, and concomitantly with the progression of synapsis, SA1 forms discrete linear threads resembling the initial SC stretches between homologous chromosomes (Figure 3C and 3D). Accordingly, pachytene spermatocytes display multiple stained linear structures over the chromatin, with their ends close to the nuclear periphery (Figure 3A–3D). After 3-D reconstruction of the nuclei, using either confocal or optical sections, it is obvious that in each spermatocyte there are 11 fluorescent lines that correspond to the number of bivalents of the species analyzed. The ends of these linear SA1 structures, which locate in close proximity to the nuclear periphery, present two separate expansions (Figure 3E–3G). In a frontal view, these signals appear as two well-defined dots (Figure 3F). Therefore, these structures may represent the attachment plates of the LEs of the SC that associate with the nuclear envelope. It is worth mentioning that the single AE of the X chromosome is never visualized after SA1 detection and 3-D reconstructions (Figure 3H–3J). On these grounds, our results indicate that SA1 is only located on those chromosomal regions that seems to achieve SC development.

### SA1 Persists in Desynapsed Bivalents

In diplotene spermatocytes, the well-defined SA1 lines become irregular and with lateral excrescences along their length, except at the association plates (Figure 3K and 3L). At diakinesis, SA1 labeling is confined to the interchromatid domain and begins to be detectable in the centromeric region of all bivalents (Figure 3M and 3N).

### RAD21 and SA1 Present Similar Expression Patterns during the First Meiotic Division

**Figure 4:**
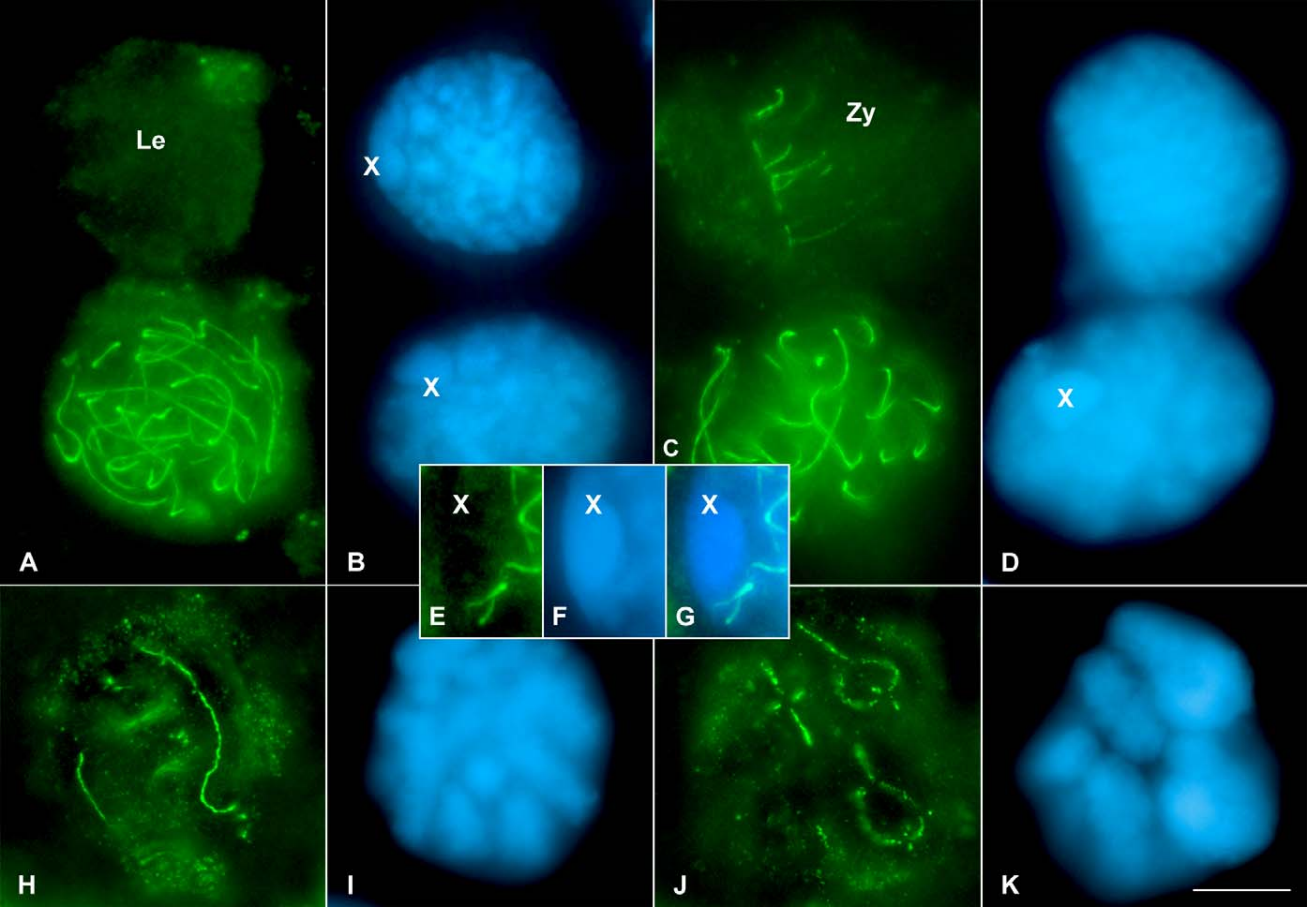
Localization of RAD21 in the Grasshopper L. migratoria Spermatocytes throughout First Meiotic Prophase (A and B) Comparing the two spermatocytes of the image, one in leptotene and the other one in pachytene, the absence of RAD21 signaling in leptotene is clearly seen. In the pachytene spermatocyte, RAD21 conforms to linear structures that resembled the SC position. LE, leptotene. (C and D) Zygotene and pachytene spermatocytes. Note the presence of short RAD21 threads in zygotene, which correspond to the base of the bouquet configuration. Zy, zygotene. (E–G) Enlarged images of the optical sections throughout the whole sex chromosome after RAD21 staining (E), DAPI counterstaining (F), and their merged image (G). The absence of RAD21 signaling inside the sex chromatin is evident. (H and I) Diplotene spermatocyte in which the irregular appearance of the RAD21 signal is evident. (J and K) Diakinesis spermatocyte showing the location of RAD21 at the interchromatid domain. (B, D, I, and K) correspond to the DAPI-stained chromatin of the spermatocytes.

To determine the distribution of RAD21 in prophase I spermatocytes, we performed the immunolocalization of RAD21 in grasshopper squashed spermatocytes (Figure 4). Leptotene spermatocytes do not show any RAD21 signals inside the nuclei (Figure 4A and 4B), but, in contrast to SA1, RAD21 is not detected in the periphery of the nucleus and is also undetectable in nucleoli. Afterward, by zygotene, short discrete stretches of RAD21 are observed in certain nuclear regions (Figure 4C and 4D). At pachytene, linear RAD21 structures, with both of their tips ending in the nuclear periphery, are visualized (Figure 4A–4D). After analyzing the 3-D reconstruction of these spermatocytes, it can be concluded that the number and size of these lines correspond to the number and size of the bivalents of the species (Figure 4A–4D and Video S9). Like SA1, and in clear contrast to SMC3, no threads of RAD21 are detected inside the sex chromosome chromatin (Figure 4E–4G and Video S9). A barbed wire–like localization of RAD21 is observed at diplotene (Figure 4H and 4I), which afterward localizes at the interchromatid domain by diakinesis (Figure 4J and 4K). These results indicate a similar spatio-temporal expression pattern of RAD21 and SA1 cohesin subunits during the first meiotic prophase in the two grasshopper species analyzed.

### Different Cohesin Subunits Locate at the Same Place but Do Not Fully Colocalize

**Figure 5:**
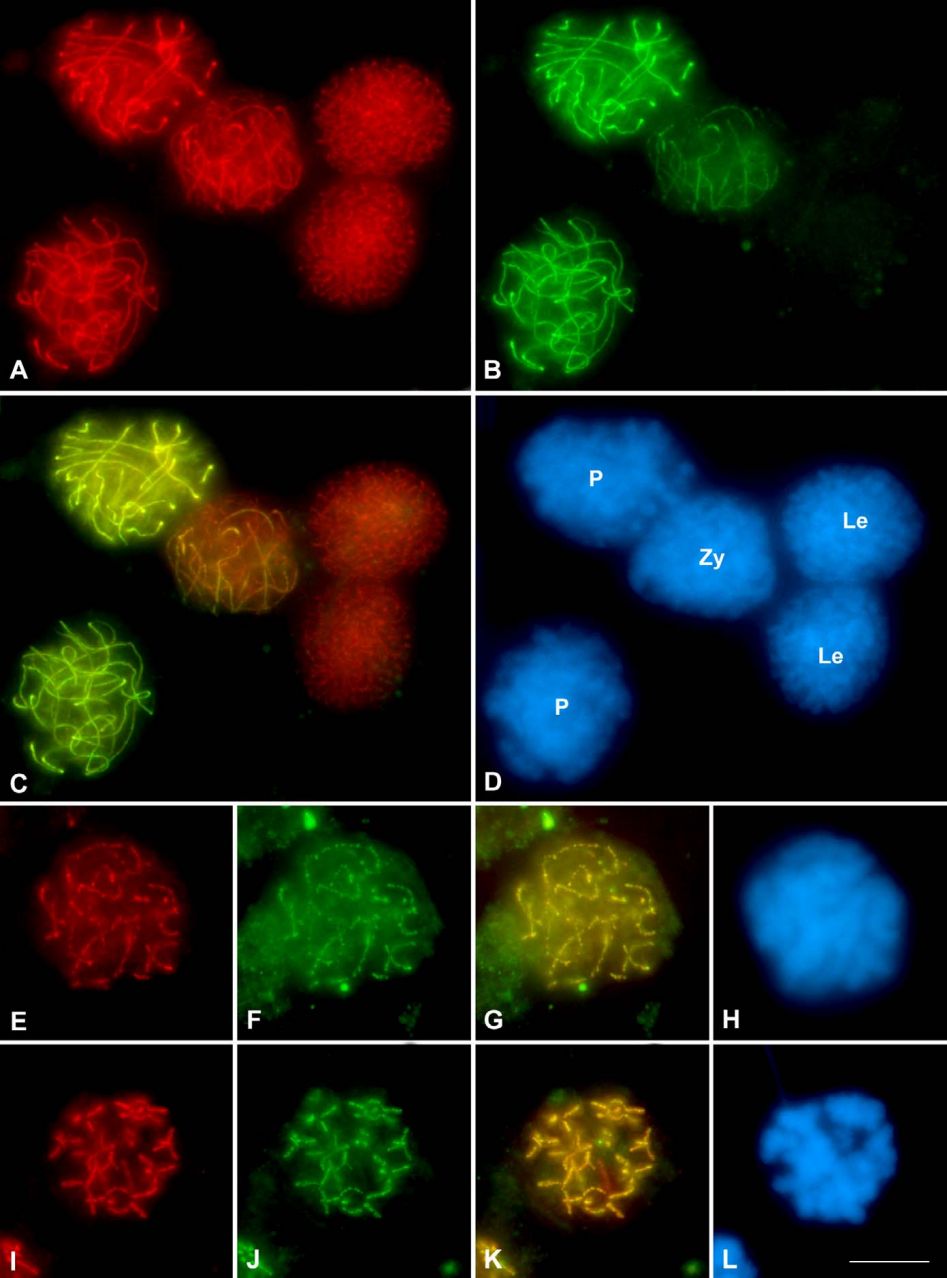
Double Immunolocation of RAD21 and SMC3 in the Grasshopper L. migratoria Spermatocytes during First Meiotic Prophase In all of the images, SMC3 is presented in red, RAD21 in green, and in the superimposed images the colocalization regions are visible in yellow. The chromatin was counterstained with DAPI (blue). (A–D) Panoramic vision of different spermatocytes in leptotene, zygotene, and pachytene. The SMC3 labeling is presented in (A), the RAD21 in (B), their superimposition in (C), and finally in (D), the DAPI staining of the chromatin. Note the complete colocalization of SMC3 and RAD21 at the synapsed autosomal regions in both zygotene and pachytene spermatocytes. Le, leptotene; Zy, zygotene; P, pachytene. (E–H) From early up to (I–L) late diplotene the colocalization detected in pachytene is gradually lost.

A double immunolocalization of SMC3 and RAD21 was performed in order to analyze their relative chromosomal distribution during prophase I (Figure 5). Whereas SMC3 reveals the AEs in leptotene spermatocytes, no RAD21 signal was evident in these nuclei (Figure 5A–5D and Video S10). Zygotene spermatocytes are characterized by the colocalization of both cohesin subunits in the synapsed chromosomal regions (Figure 5A–5D and Video S10). In contrast, the unsynapsed regions are only stained by the anti-SMC3 antibody (Figure 5A–5D and Video S10). A complete colocalization of both cohesin subunits is clearly apparent in pachytene (Figure 5A–5D and Video S10). These results were obtained and validated by both optical sections, under fluorescence microscopy (Figure 5), as well as by sequential scanning capture of images under confocal microscopy (unpublished data). Surprisingly, the complete colocalization pattern of these proteins is not maintained at diplotene and diakinesis (Figure 5E–5L and Video S11) since, in addition to the yellow-labeled regions that indicate colocalization, we can also observe a few chromosomal regions where only SMC3 (red-labeled regions) or RAD21 (green-labeled regions) labeling is present (Figure 5G and 5K).

## Discussion

### SMC3 Is Located at the Base of Chromatin Loops throughout the First Meiotic Prophase

**Figure 6:**
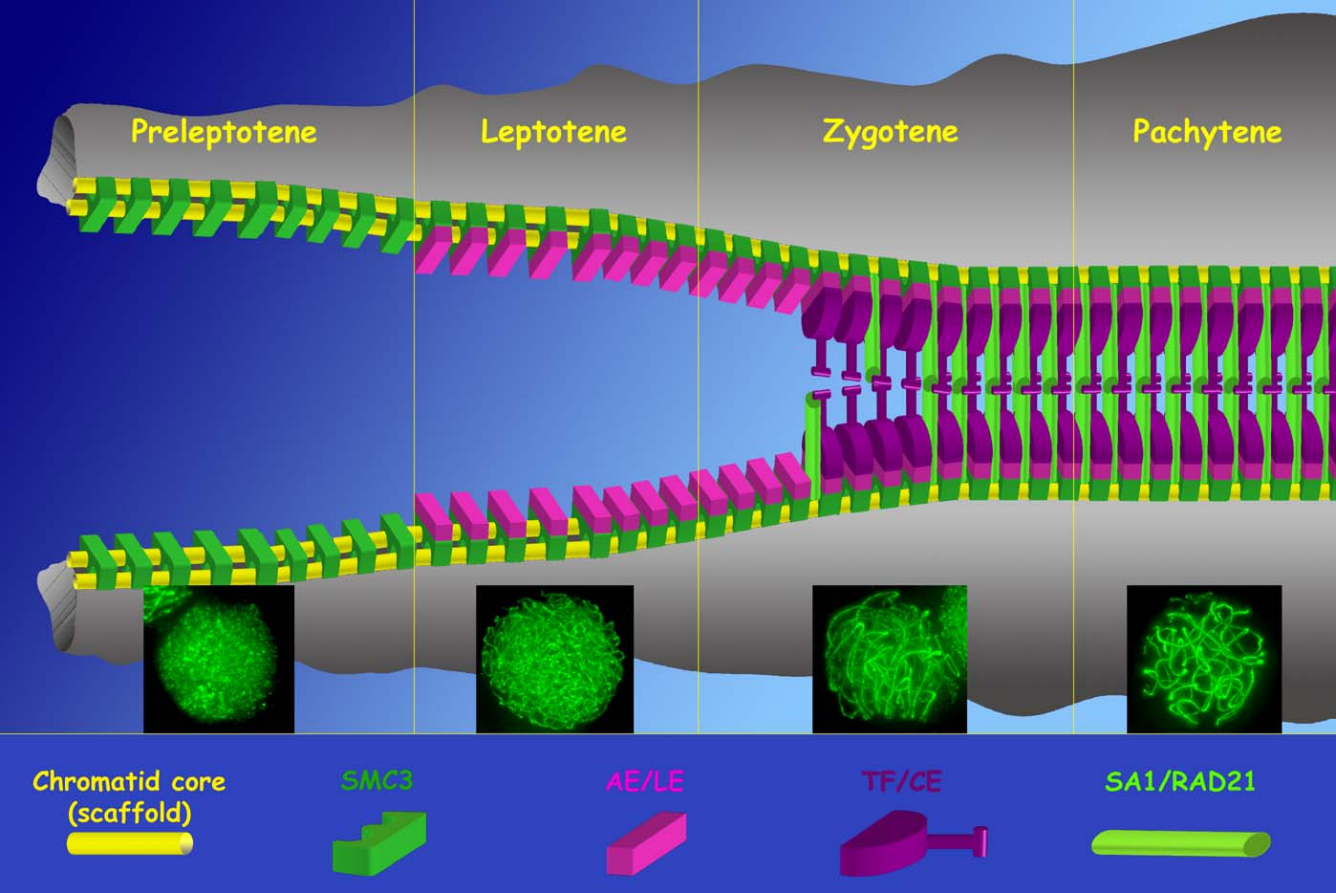
Schedule of Homologous Chromosome Synapsis Possible interrelation of the different elements implied in the meiotic chromosome organization throughout the first meiotic prophase. In the schematic representation, the chromatid axes, the synaptonemal complex components, and some subunits of the cohesin complex are outlined in different colors. Note the interrelationship among the different elements depicted, and the temporal separation in the assembly of different cohesin subunits. For more details see the text.

SMC1 and SMC3 are involved in sister chromatid cohesion during meiosis and also seem to be essential for the organization of the AE structure in which chromatin loops are attached in mammal meiosis [46]. A participation of SMC3 in the structure of the LEs of *Drosophila* has also been proposed [47]. In the two grasshopper species analyzed, we have detected the presence of SMC3 in all prophase I stages, whereas in preleptotene cells there is no specific nuclear distribution of this protein (similar to that found in spermatogonial interphases). At the onset of prophase I in grasshoppers, SMC3 re-localizes in well-defined and continuous lines; this situation is slightly different from that previously reported in mammals since it has been demonstrated that, at least in spreads, both proteins localize in a beaded structure along the AEs/LEs at pachytene [46]. Therefore, the morphology of these lines and their development throughout prophase I lead us to propose that SMC3 is closely associated to the AEs/LEs at the base of the chromatin loops (Figure 6). This assertion, in agreement with mammal observations, is reinforced by the correspondence between the number and size of the SMC3 signals at pachytene and the number of SCs observed in the two species studied.

Therefore, although in grasshoppers it is not possible to assay the SC formation directly; it could be inferred from the identification of thin and thick SMC3 filaments that correspond to synapsed and unsynapsed regions, respectively. The morphology of the SMC3 axis and its dynamics allow an accurate identification of the different prophase I stages, and, a subsequent analysis of the presence and localization of other proteins throughout prophase I [43,48,49].

### Sequential Loading of Cohesins SMC3, SA1, and RAD21

The cohesin complex is responsible for the maintenance of sister chromatid cohesion in both mitotic and meiotic divisions. However, its composition and dynamics seem to be different in both processes. Thus, while in mitosis sister chromatid cohesion is released in each cellular division, cohesion in meiosis is lost in the two temporally separated divisions: arm cohesion at anaphase I and centromeric cohesion at the onset of anaphase II [45].

We have demonstrated here that a differential loading of cohesin subunits takes place during prophase I in grasshoppers. In this sense, whereas SMC3 labeling is already present in preleptotene cells, SA1 and RAD21 are not detectable until the initiation of synapsis at zygotene. Likewise, the double immunolocalization of SMC3 and RAD21 clearly defines a sequential association of these proteins to meiotic chromosomes. A possibility that cannot be excluded is that a small amount of the cohesin subunits, SA1and RAD21, is already associated with the chromosome at premeiotic S phase, but their amounts are not enough to be detected by the immunolocalization protocol used in this study. However, since we are able to clearly visualize the localization of SMC3 from early meiotic prophase I onward, and considering that SA1 and RAD21 participate within the same cohesin complex with SMC3 in a similar stoichiometry, we should have necessarily detected them. This timing of appearance of SMC3 versus SA1 and RAD21, and its localization pattern and dynamics during the first meiotic prophase, also concurs with previous biochemical results from *Drosophila* embryos, where the immunoprecipitation experiments of RAD21 intriguingly suggest that RAD21 and SA1 are more tightly associated to each other than they are with the SMC subunits of the cohesin complex [50]. However, after the coimmunoprecipitation assays using grasshopper testis nuclear protein extracts, we cannot discern whether RAD21 and SA1 are part of another cohesin complex, or, on the contrary, whether or not there is a sequential addition of the cohesin subunits.

Since SA1 and RAD21 cohesin subunits only became detectable at zygotene, we speculate that following the initial association of the canonical cohesin complex to the chromatin in early meiosis, a second round of cohesin loading, which included at least SA1 and RAD21, could be necessary to increase the bivalent stability. This additional round of cohesion establishment may be necessary to reinforce the arm cohesion of bivalents, in order to counteract the polar forces throughout congression, during prometaphase I and metaphase I, until the segregation of recombined homologous chromosomes at anaphase I. In this sense, the subsequent loading of both RAD21 and SA1 at zygotene is consistent with the necessity of arm cohesion reinforcement in order to prevent a premature separation of homologs prior to anaphase I.

The differential loading of cohesin subunits has indirectly been suggested in other organisms. For instance, in mammals it has been proposed that REC8 may provide a basis for AE formation in early prophase I prior to the appearance of SMC1β and SMC3 [51]. On the other hand, the depletion of TIM-1 in Caenorhbditis elegans (clock protein TIMELESS in *Drosophila*) prevents the assembly of non-SMC subunits onto meiotic chromosomes. However, a cohesin complex with SMC components is already loaded, suggesting that SMC1 and SMC3 are associated onto chromatin independently of non-SMC proteins [52]. Finally, in *Arabidopsis,* the protein SWI1, which appears to be required for early meiotic events that are at the crossroad of sister chromatid cohesion, is expressed exclusively in meiotic G1 and S phase [53]. Other cohesin proteins, such as REC8 and SCC3, will appear later [54].

In grasshopper spermatocytes, the single X chromosome shows an AE that never appears partially or fully synapsed at prophase I [44]. Consequently, the study of this chromosome is of particular interest as regards the composition of its cohesin axis. Our current results undoubtedly demonstrate that in prophase I spermatocytes, a single SMC3 axis is always observable inside the sex chromosome chromatin. By contrast, whereas both SA1 and RAD21 are present in each autosomal bivalent from zygotene onward, neither SA1 nor RAD21 are detected in the X chromosome throughout prophase I. In strong agreement with our finding that RAD21 and SA1 are only present in those autosomal regions that have achieved synapsis, the special features of the X chromosome emphasize our previous assertion that only when homologous synapsis progresses, the RAD21 and SA1 proteins are loaded onto chromosomes. Whether RAD21 and SA1 participate in a distinct cohesin complex other than SMC3, or whether our observations only indicate a sequential addition of cohesin subunits in meiotic bivalents, or both, still remains an open question. However, the bulk of these data taken together strongly suggests that, as in other organisms, different cohesin complexes may coexist during meiosis in grasshoppers, and that the presence of distinct cohesin complexes may contribute to the different dynamic meiotic chromosome requirements.

### Working Model

JSR and coworkers [55] studied the chromosome organization in grasshopper spermatocytes by light microscope analysis of silver-stained cores and concluded that the chromatid core represents the scaffold of each sister chromatid. Afterward, it was proposed as a model of meiotic chromosome organization (based on light and electron microscope observations), where the chromatid core locates at the base of the chromatin loops, would act as the framework for the further assembly of the AE/LE proteins [56]. Here, we incorporate the contribution of the subunits of the cohesin complex (Figure 6). Some of these subunits, like SMC3, could be considered as a structural component of an initial cohesin axis (dark green in Figure 6), which may in fact be the real framework for the assembly of the AE/LE proteins (pink), and that is closely associated to the chromatid cores (yellow). The subsequent association of the AE proteins to this primary cohesin axis would represent the cytologically detected AEs. Simultaneous with the assembly of the transverse filament/central element protein/s (purple) at zygotene, a second cohesin subunit loading containing at least SA1 and RAD21 (light green) would occur just before, during, or immediately after homologous synapsis. At pachytene, when the tripartite structure of SCs is fully formed, not only the SC proteins, but also cohesin complexes, would contribute essentially to the maintenance of the correct association of homologous chromosomes. This model of meiotic chromosome organization provides new essential insights to explain both the existence of synapsis in the absence of SYCP3 [57] and the existence of homologous chromosome alignment in the SYCP1-deficient mouse model [58]. Furthermore, observations of later meiotic stages in grasshopper spermatocytes, from diplotene up to telophase I, indicate that the different cohesin subunits do not fully colocalize in these stages (JSR, unpublished data).

## Materials and Methods

### Materials.

Adult males of the grasshopper species E. plorans and *L. migratoria,* (Orthoptera: Acrididae) collected in natural populations, or bred in the laboratory, were used in the present study. *Drosophila* S2 cells were grown in Schneider's medium (Sigma, http://www.sigmaaldrich.com) supplemented with 10% fetal bovine serum at room temperature. Adult male C57BL/6 mice from our animal facilities were also used for this study.

### Primary antibodies.

To detect the cohesin subunit SMC3, we employed a polyclonal rabbit anti-SMC3 antibody (AB3914; Chemicon International, http://www.chemicon.com) raised against a synthetic peptide from human SMC3. It is worth noting that only the lot number 220701985 of the cited antibody rendered tiny immunolabeling signals in two grasshopper species contrary to the actually commercialized stock provided by Chemicon. SA1 was detected by a rabbit anti-DSA1 antibody generated against *Drosophila* SA1 recombinant protein [59]. RAD21 was detected using a rabbit anti-DRAD21 antibody raised against a bacterially expressed carboxy-terminal fragment of *Drosophila* RAD21 [60].

### Immunoblotting.

Testes from adult E. plorans and L. migratoria males were removed and placed in 1 ml of 0.5% Triton X-100 in PBS for 5 min and then washed with PBS, as previously described [48]. Schneider cells (5 × 10^6^ cells) were harvested and washed with PBS. Testes from adult male C57BL/6 mice were removed and processed as previously described [61]. Nuclear extracts from grasshopper testes and Schneider cells were obtained using the NE-PER Nuclear and Cytoplasmic Extraction Reagent Kit (Pierce, http://www.piercenet.com) according to the manufacturer's instructions. For Western blotting, proteins were resolved by 8% SDS-PAGE [62] and blotted with the following antibodies diluted in 4% nonfat dry milk in PBS: anti-DSA1 at a 1:1,000 dilution, anti-DRAD21 at a 1:500 dilution, and anti-SMC3 at a 1:5000 dilution.

### Immunofluorescence microscopy.

Testes were removed and fixed for immunofluorescence as previously described [42]. Briefly, testes were fixed in freshly prepared 2% formaldehyde in PBS containing 0.1% Triton X-100 (Sigma). After 5 min, several seminiferous tubules were placed on a slide previously coated with 1 mg/ml poly-L-lysine (Sigma) with a generous drop of fixative, and tubules were gently minced with tweezers. After exerting pressure on the cover slip, slides were frozen in liquid nitrogen and the cover slip removed with a razorblade. The slides were then rinsed three times for 5 min in PBS and incubated with the corresponding primary antibody for 45 min at room temperature, or overnight at 4 °C. Primary antibodies diluted in PBS were used at the following dilutions: anti-DSA1 antibody at 1:50 dilution, anti-DRAD21 antibody at 1:50 dilution, and anti-SMC3 at a 1:30 dilution. Following three washes in PBS, the slides were incubated for 30 min at room temperature with a fluorescein isothiocyanate-conjugated goat anti-rabbit IgG (Jackson ImmuoResearch, http://www.jacksonimmuno.com) secondary antibody at a 1:150 dilution in PBS. In the double immunolabelling experiment with SMC3 and RAD21, since the two primary antibodies were generated in the same host species, we proceeded as previously described [63]. Subsequently, slides were rinsed in PBS and counterstained for 3 min with 5 μg/ml DAPI (4′,6-diamidino-2-phenylindole). After a final rinse in distilled water, slides were mounted with Vectashield (Vector Laboratories, http://www.vectorlabs.com) and sealed with nail varnish.

Observations were performed using an Olympus BX61 (http://www.olympus.com) microscope equipped with a motorized Z-axis and epifluorescence optics. The images were captured with a DP70 Olympus digital camera using the associated analySIS software (Soft Imaging System, Olympus). Samples were also analyzed under different confocal laser scanning microscopes, a Leica TCSNT (http://www.leica.com), a Bio-Rad radiance 2000 (http://www.bio-rad.com), and an Olympus IX-70 inverted microscope equipped with a confocal laser scanning system (Fluoview 300). Images were captured by sequential scanning, noise-filtered, corrected for background, and processed using the appropriate software. Images were finally analyzed and processed using Adobe Photoshop 6.0 software (http://www.adobe.com), the public domain software ImageJ (National Institutes of Health, United States; http://rsb.info.nih.gov/ij), and VirtualDub (VirtualDub, http://www.virtualdub.com).

## Supporting Information

Video S1 Interphase Spermatogonia This video, as well as Videos S2–S8, corresponds to the 3-D reconstructions of the different grasshopper cells included as Z-projections in Figure 2. For the correct visualization of the videos, please click the loop/boucle option in your video player before running the videos.
(1.4 MB MOV)

Video S2 Preleptotene (652 MB MOV)

Video S3 Leptotene (1.3 MB MOV)

Video S4 Onset of Synapsis at Early Zygotene (1.2 MB MOV)

Video S5 Bouquet Formation in Zygotene (1.2 MB MOV)

Video S6 Pachytene (1.5 MB MOV)

Video S7 Early Diplotene (884 KB MOV)

Video S8 Late Diplotene For the correct visualization of the videos, please click the loop/boucle option in your video player before running the videos.
(928 KB MOV)

Video S9 3-D Reconstruction of the RAD21 Cohesin Subunit in a Grasshopper Pachytene Spermatocyte The chromatin is in blue, whereas RAD21 is in green. Note the absence of labeling inside the whole domain occupied by the sex chromosome (region of condensed chromatin in the nuclear periphery located at the top of the video). For the correct visualization of the video, please click the loop/boucle option in your video player before running the video.
(1.9 MB MOV)

Video S10 This Video Corresponds to the Same Cells Z-Projected in Figure 5A–5C Double immunolocation of the cohesin subunits SMC3 (red) and RAD21 (green). The colocalization regions are seen in yellow. The different prophase I stages are labeled in this video. For the correct visualization of the video, please click the loop/boucle option in your video player before running the video.
(1.9 MB MOV)

Video S11 Different Prophase I Cells Double immunolocation of the cohesin subunits SMC3 (red) and RAD21 (green). The colocalization regions are seen in yellow. The different prophase I stages are labeled in this video. Note the absence of full colocalization in the diplotene spermatocyte located in the middle of the video. For the correct visualization of the videos, please click the loop/boucle option in your video player before running the videos.
(1.9 MB MOV)

## Abbreviations

AE: axial element
DRAD21: *Drosophila* RAD21 cohesin subunit
DSA1: *Drosophila* SA1 cohesin subunit
LE: lateral element
RAD: radiation-sensitive mutant
REC: meiotic recombination protein
SA1: Stromal antigen protein 1
SC: synaptonemal complex
SCC: sister chromatid cohesion protein
SMC: structural maintenance of chromosome protein
